# Thalidomide in the treatment of erythema nodosum leprosum (ENL) in an outpatient setting: A five-year retrospective analysis from a leprosy referral centre in India

**DOI:** 10.1371/journal.pntd.0008678

**Published:** 2020-10-09

**Authors:** Brahmaiah Upputuri, Michael Sukumar Pallapati, Patrick Tarwater, Aparna Srikantam

**Affiliations:** 1 Clinical Division, LEPRA Society-Blue Peter Public Health and Research Centre (BPHRC), Hyderabad, India; 2 Department of Epidemiology, Johns Hopkins Bloomberg School of Public Health, United States of America; 3 Clinical and Laboratory Research Division, LEPRA Society-Blue Peter Public Health and Research Centre (BPHRC), Hyderabad, India; National Hansen's Disease Programs, UNITED STATES

## Abstract

Erythema nodosum leprosum (ENL), or type 2 lepra reaction, is a multi-system immune-mediated complication in patients with multibacillary leprosy, frequently associated with chronicity and recurrences. Management of ENL requires high doses of oral corticosteroids, which may not be universally effective and pose serious adverse effects. Thalidomide has proven to be a steroid-sparing agent and is useful in controlling the reactions. However, many centres do not employ it in outpatient settings due to adverse effects and teratogenicity risk. Hence, we studied the feasibility of treating ENLs and reported the therapeutic outcome.This is a five-year record-based analysis of ENL leprosy patients treated with thalidomide, includingdescriptive statistics of demographic variables. Clinical characteristics were stratified by treatment compliance status (yes/no). Incidence rates and rate ratios for recovery stratified by bacillary index, type of ENL presentation and MDT treatment status were calculated.Out of 102 ENL patients treated with thalidomide, 68 (66.7%) were compliant and improved. Among them, ENL recurrence was noted in 11(16.2%) patients. The commonest thalidomide side effect was pedal oedema (73.5%). Patients with bacillary index (BI) less than or equal to 4.0 had a 37% increase in the incidence of recovery. Patients with acute ENL were almost twice as likely to recover as those with chronic ENL. Also, the improvement was two and a half times greater among those who completed MDT as compared to those on MDT. The study showed that thalidomide treatment for patients with ENL is possible in outpatientclinics. We also successfully prevented pregnancies to a larger extent through counselling for contraception.We observed that early institution of thalidomide induces faster remission and prevents ENL recurrence.

## Author summary

Erythema nodosum leprosum (ENL), is an immune-mediated condition of leprosy.It is a multi-system disorder which can occurbefore, during or after completion of multidrug therapy for leprosy.ENL is often characterised by chronicity and recurrence. Management of ENL requires long-term oral corticosteroids which are associated with adverse effects.Thalidomide is an alternative to steroids, but many centres donot use it in their outpatient clinics due to the significant concern of drug-producing birth defects.This study provides additional evidence of the feasibility of thalidomide treatment in ENL at outpatient settings.

## Introduction

In India, leprosy remains a public health problem, contributing more than half of the global burden [1]. It is caused by *Mycobacterium leprae*, which mainly affects the skin and peripheral nervous system [2].Multi-drug therapy (MDT) is highly effective in the treatment of infection; however, a significant proportion of patients still developreactions [3]. There are two major types of leprosy reactions: Type 1 reaction (T1R) is a type IV hypersensitivity reactionseen in borderline leprosy patients,causing inflammation in pre-existing skin lesions and/or neuritis [4], and Type 2 reaction (T2R), also known as erythema nodosum leprosum (ENL), is an immune complex-mediated complication of lepromatous leprosy (LL) thatusually occurs within 6 months of MDT initiation but can occur before, during or after MDT [5]. Patients develop erythematous, tender, painful nodules, which arise in crops lasting for several days along with constitutional symptoms and heal with post-inflammatory hyperpigmentation; less commonly, they may present as pustular, bullous, ulcerated, and erythema multiforme-like lesions [6, 7]. These lesions are commonly seen on the face and extensor aspects of limbs. Iridocyclitis, orchitis, arthritis, dactylitis, lymphadenitis and nephritisare other documented complications of ENL[3].Lepromatous leprosy or a bacillary index (BI) over 4 are the proven risk factors [5].

ENL is a medical emergency and is treated with anti-inflammatory drugs and immunomodulators as it hasa high potential to cause disabilities. The disabilities are due tonerve damage with decreased peripheral nerve function because of motor and sensory loss [8]. Corticosteroids are the mainstay treatment to relieve symptoms [9, 10], thus improving the quality of life. Corticosteroids are used to treat active ENL, but they cannot prevent ENL recurrences, and their continuous usage is associated with complications, including hypertension, diabetes, impaired wound healing and increased risk of infections.They are also responsible for seriouscomplications, such as gastrointestinal ulcers and osteoporosis [11]. ENL lesions may recur episodically or become chronic and may even persist over many years [12]. Most chronic and recurrent ENL patients are treated with long periods of steroids that commonly lead to steroid dependence. This mandates the need for an alternative to steroidswith fewer side effects to treat ENL symptoms. A study in Ethiopia showed that there was a 9% mortality in patients with ENL being treated with steroids [3].

Thalidomide is highly effective in the treatment of patients with chronic or recurrent ENL. It acts by inhibition of selective gene expression of tumour necrosis factor-α (TNF-α) involved in the pathogenesis of nerve damage in leprosy and other mechanisms contributing to its anti-inflammatory effect [13,14].A recent study found a significant reduction in steroid-induced adverse effects after thalidomide treatment [11].Thalidomide is considered an effective treatment for ENL by the World Health Organisation (WHO) Expert Committee,but there are reports stating non-recommendation of thalidomide, which contradicts the published evidence [9, 11]. However, its availability is a major concern due to cost and teratogenic risk, and there is a risk of development of peripheral neuropathy [9]. Cyclosporine, pentoxifylline,methotrexate and azathioprine are advocated as alternatives to thalidomide but is unclear to choose the preferred drug, and they are not very effective in patients with severe episodes of prolonged reactions [9,14]. Many centres donot use thalidomide in outpatient clinics because they are worried about adverse effects; importantly, the drug is a well-known teratogen and has the potential in inducing a range of birth anomalies, most commonly limb defects [15]. Hence, pregnancy prevention is of utmost importance.

We describe our experience of the use of thalidomide in an outpatient set-up in the management of ENL.

## Methods

### Ethics statement

The study protocol was approved by the institutional ethics committee of LEPRA Society-Blue Peter Public Health and Research Center (BPHRC), Hyderabad, India. All the participants were adults and had received thalidomide treatment after obtaining written informed consent and thorough counselling to avoid pregnancy.

### Patients

The database of ENL patients treated with thalidomide in the outpatient department of LEPRA society BPHRC, Hyderabad, was reviewed for five years between 2010 to 2015.Data were collected on all ENL patients for demographic details, disease-specific parameters, clinical course, treatment compliance, adverse events and treatment outcome, including death. Patients were started on thalidomide after providing written, informed consent and receiving counselling by the following system for thalidomide education and prescribingsafety (STEPS) protocol [16]. Women in the reproductive age groupwere tested for pregnancy before the start of the drug and counselled to adopt dual contraceptive methods comprising of a highly effective method along with a barrier method of ≥4 weeks before, throughout and for 4 weeks after the end of therapy. Male patients were also advised to use a barrier contraceptive method at the initiation of treatment and up to 1 month after the completion of treatment. Patients were prescribed a 4-week course of the drug during each visit. They were also instructed not to share the drug. Routine lab investigations, full blood count, urine examination and renal function tests wereperformed periodically. All patients were followed-up for five years of post thalidomide treatment. Definitions and types of ENL were taken according to ENLIST consensus, and applied while analysing the data as shown below [17].

### Definitions

Acute ENL: a single episode lasting less than 24 weeks.

Recurrent ENL: a second or subsequent episode of ENL occurring 28 days or more after stopping treatment for ENL.

Chronic ENL: ENL occurring for 24 weeks or more during which a patient has required ENL treatment either continuously or where any treatment-free period had been 27 days or less.

Steroid dependence: recurrence of symptoms upon discontinuation of steroids or even when steroid doses are tapered gradually [11].

Hypertension:defined as systolic blood pressure (BP) of at least 140 mm Hg or diastolic BP of at least 90 mm Hg

Diabetes: defined as having a high fasting plasma glucose reading (≥126mg/dL [7.0mmol/L] or ≥200 mg/dL [11.1 mmol/L] if patients reported not fasting

Complete recovery:defined as a complete resolution of symptoms (cutaneous as well as systemic) upon treatment.

Treatment compliance: adherence to the complete course of thalidomide treatment

### Thalidomide protocol

The organization has an existing protocol for thalidomide administration in the management of ENL, considering the severity of symptoms. Dosage protocol followed was 300mg once daily (OD) for 1 month, 200mg OD for 1 month and 100mg OD for one month.Then, the patients were maintained on 50mg of thalidomide per day until complete remission and tapered. The patients were encouraged to take the drug in the night to minimise sedation. The patients returned every month for follow-up and were monitored for adverse events if any, and disease progression.The steroid dosage will be tapered according to WHO guidelines.Whenever patients hada flare-up of ENL while on thalidomide therapy,theywill be treated by increasing the thalidomide dose for a few weeks. Thalidomide was provided free of cost.

Response to thalidomide was assessed by complete patient recovery and measuring further ENL episodes.

### Statistical methods

Summary statistics are presented for demographic variables, for clinical characteristics of participants at baseline (i.e., initiation of thalidomide) and clinical response after treatment initiation in separate tables, respectively. Descriptive statistics for variables measured on a continuous scale are presented as means and standard deviations (sd) and/or medians and 25th and 75th percentiles (25th, 75th). For variables measured on a categorical scale, descriptive statistics are presented as column percentages of the column total frequency. All summary statistics are presented for all participants and by ENL recovery status. Time to recovery analysis presented person time in months, and incidence rates as descriptive statistics for compliance status, thalidomide treatment indication and ENL presentation status were also analysed. Data was entered, managed and stored in MS Excel (Microsoft Office), and data analyses were conducted in Stata 14 (Stata Corp).

## Results

During the study period, 225 leprosy patients with ENL were registered for the treatment. Of them, 102 (45.3%) patients were treated with thalidomide; 68 (66.7%) were compliant and improved while taking thalidomide. As shown in Table 1, the patients were approximately 34 years old, 70% were males, and 80% were married; 41.2% patients had high BI (greater than 4.1), and 58.8% had low BI (less than or equal to 4.0).About two-thirds of the patients presentedwith lepromatous leprosy and the remainder with borderline leprosy. Table 1 also shows these demographic variables stratified by thalidomide compliance status.

**Table 1:** Demographic summary statistics among 102 ENL patients initiating thalidomide by treatment compliance.

Demographic Variables	Total	Treatment Compliance	
		No	Yes
Number of Patients	102	34	68
Age, mean (std)	34.4 (11.5)	35.9 (12.8)	33.6 (10.8)
Gender, %			
Male	69.6	64.7	72.1
Female	30.4	35.3	27.9
Marriage Status, %			
No	19.9	14.7	22.1
Yes	80.4	85.3	77.9
Median Bacillary Index			
Mean (std)	3.59 (1.47)	3.65 (1.54)	3.56 (1.39)
< = 4.0, %	58.8	55.9	60.3
4.1, %	41.2	44.1	39.7
Ridley-Jopling Classification, %			
Borderline lepromatous, BL	36.3	35.3	36.8
Lepromatous, LL	63.7	64.7	63.2

Table 2 presents the descriptive statistics of the clinical characteristics for the thalidomide-treated ENL patients stratified by compliance status. Almost 90% of the patients started thalidomide treatment because of steroid dependency. In addition, over 80% presented with classical ENL, specifically, 13% acute, 25% recurrent and 44% chronic. On average, patients had approximately 1 year of ENL when started on thalidomide, and 32% had completed MDT treatment. Twenty-two percent of the study cohort had associated comorbidities, including diabetes, asthma, tuberculosis, and hypertension.

**Table 2:** Clinical characteristics summary statistics among 102 ENL patients initiating thalidomide by treatment compliance.

Clinical Characteristics	Total	Treatment Compliance	
		No	Yes
Number of Patients	102	34	68
Thalidomide indication, %			
Steroid Dependency	89.2	85.3	91.2
Steroid Toxicity	7.8	11.8	5.9
Contraindication	2.9	2.9	2.9
Duration of Thalidomide use, months			
mean (sd)	9.4 (8.6)	4.8 (3.6)	11.7 (9.4)
ENL Presentation and Type, %			
Classical			
Acute	12.8	14.7	11.7
Recurrent	25.5	20.6	27.9
Chronic	44.1	41.2	45.6
Ulcerative	17.6	23.5	14.7
Duration of ENL, months			
Mean (std)	14.2 (11.0)	14.6 (11.0)	14.0 (11.1)
Median (25^th^, 75^th^)	12 (5,24)	12 (6,24)	12 (4, 22)
MDT Status, %			
Initial	8.8	14.7	5.9
Current	58.8	58.8	58.8
Completed	32.4	26.5	35.3
Comorbidities, %			
No	77.5		
Yes	22.5		
Diabetes	7.8		
Asthma	5.9		
TB	5.9		
Hypertension	2.9		

**Fig 1:**
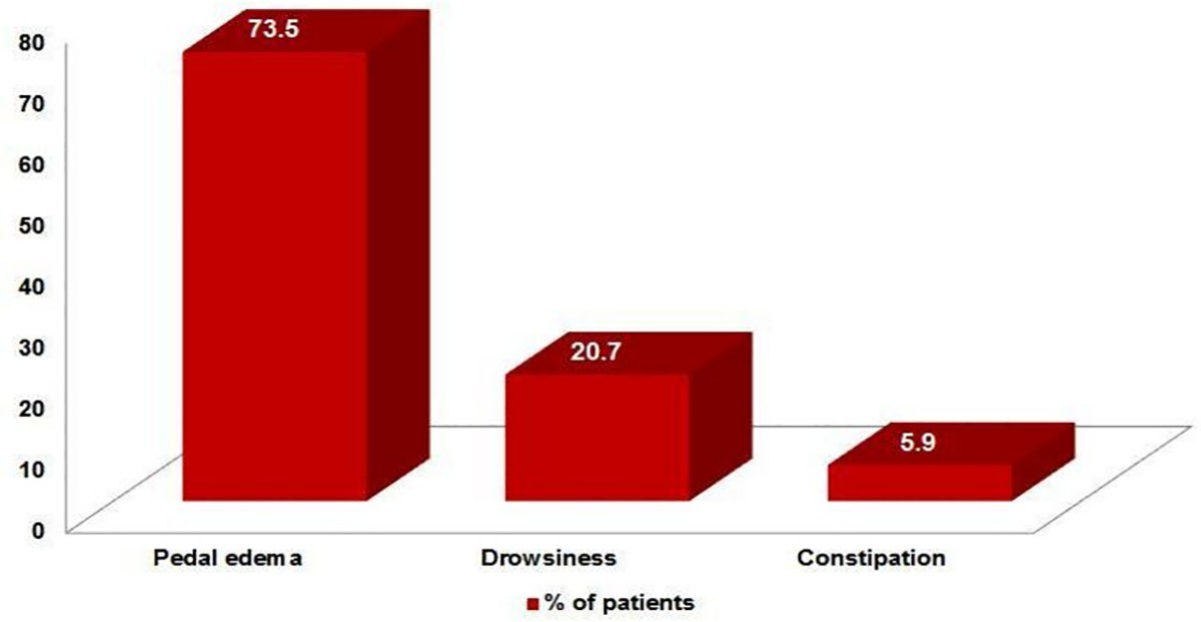
Side effects observed during thalidomide treatment.

**Fig 2:**
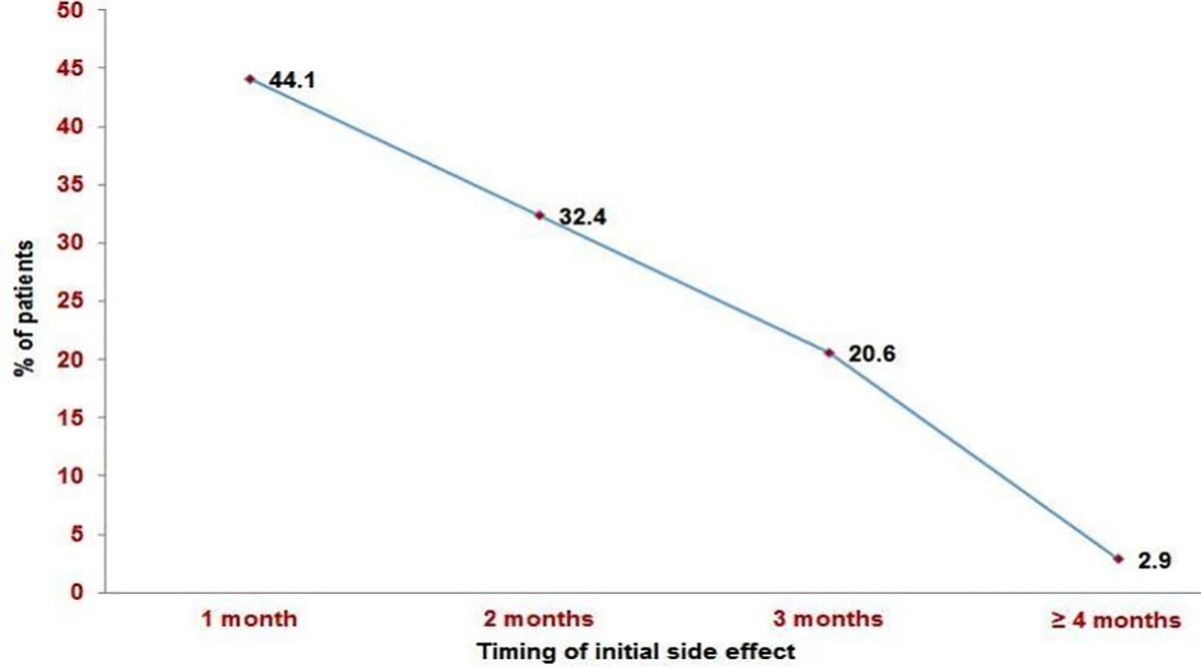
Timing of initial side effects during treatment.

Patients were on thalidomide treatment for a mean of 9 months, and34 patients discontinued after a mean of 5 months compared to over 11 months for those who recovered. Among the recovered group, ENL recurrence was noted in 16.2% of patients with a mean duration of 14.09± 3.98 months. The commonest thalidomide side effect was pedal oedema (73.5%), followed by drowsiness (20.7%) and constipation (5.9%)(Fig 1). Side effects of thalidomide treatment were observed as early as the first month of treatment (44.1%) and decreased in the subsequent months(Fig 2).

All the compliant patients recovered completely while on thalidomide therapy. Thenon-compliance among the rest was due to various reasons, such as distance, the cost involved in attending the clinic and other social factors.

The advantage of using a retrospective cohort study design is the ability to measure and utilise each patient’s time contributed during follow-up to recovery. That is, cohort studies allow the estimation of incidence rates (i.e., no. events/person-time at risk), whereby we observed ENL recovery in 68 out of the 102 (67%) patients under follow-up with an estimated incidence rate of 7.1 recoveries out of every 100 person-months of follow-up.Patients initiating thalidomide with BI less than or equal to 4.0 had a 37% increase in the incidence of recovery (e.g., time to recovery) compared to those with BI greater than 4.0. Recovery among patients presenting with acute ENL was almost 2 times greater than those with chronic ENL. Additionally, chronic ENL presenters were observed to have a 52% increase in the incidence of recovery compared to the ulcerative group. The recovery was two and a half times greater among those who have completed MDT as compared to those on MDT (Table 3). Among 68 recovered patients, 12 (17.6%) were treated with increased doses of thalidomide upon flare-up of ENL during treatment.A total of four deaths have been observed in the study (Table 4).

**Table 3:** Incidence rates and rate ratios stratified by bacillary index, type of ENL presentation and MDT treatment status.

Variables	Total	Recovery			
		Recovered, N (%)	Duration of Thalidomide use, months	Incidence Rate, per 100 person-months	Incidence Rate Ratio
Total	102	68 (66.7)	9.59	7.09	
Bacillary Index					
< = 4.0	60	41 (40.2)	5.04	8.13	1.37
> 4.0	42	27 (26.5)	4.55	5.93	1
ENL Presentation Type					
Acute	13	8 (61.5)	0.78	10.26	1.94
Recurrent	26	19 (73.1)	3.06	6.21	1.17
Chronic	45	31 (68.9)	3.86	8.03	1.52
Ulcerative	18	10 (55.6)	1.89	5.29	1
MDT treatment status					
Initial	9	4 (5.9)	0.97	4.12	1
Current	60	40 (58.8)	6.29	6.36	1.54
Completed	33	24 (35.3)	2.33	10.30	2.50

**Table 4:** Clinical details of four reported deaths observed during study follow up.

	Patient 1	Patient 2	Patient 3	Patient 4
Age, years	45	56	45	50
Gender	F	F	F	F
RJ	LL	LL	BL	LL
Comorbidities	None	Asthma	Hypertension	None
Type and duration of ENL, months	MB, 24	MB, 6	MB, 6	MB, 12
No. of ENL episodes	3	3	5	4
ENL presentation	Ulcerative	Classical	Ulcerative	Classical
Clinical events before death	Not significant	Not significant	Not significant	Cushing’s syndrome Chronic gastritis
Cause of death	Suicide, due to domestic issues	Not recorded	Hypertension	Suspected chronic adrenal suppression
Death due to ENL	No	No	No	No
Death due to steroids	No	Probable	Yes	Yes

RJ:Ridley–Jopling Classification

LL:Lepromatous leprosy

BL: Borderline lepromatous leprosy

MB:Multibacillary

ENL:ErythemaNodosum Leprosum

## Discussion

With the success of MDT in the treatment of leprosy, there is intense scientific interest in the control of leprosy reactions, especially ENL. This study describes the clinical course of ENL among the patients treated with thalidomide in an urban out-patient setting.ENL causes greater disability than underlying lepromatous disease [18]. The management of ENL aims to control inflammation and pain and prevent a recurrence. Corticosteroids are commonly employed in ENL management, and there is no alternative to steroids to bring down the symptoms quickly [11]. Steroids inhibit inflammation through various modes of action and also produce serious adverse effects. Of these, steroid dependence is particularly important and more so in ENL. It occurs due to adrenal suppression resulting from prolonged intake of steroids [19]. Each subsequent episode of ENL requires a higher dose of steroids and upon withdrawal results in recurrence. It is a challenge for the treating physician to decide minimum steroid effective dose to prevent ENL. In our study, steroid dependence was observed in 89.10% of patients in comparison to 80% in the study by J. Darlong et al.[11].

Corticosteroids are effective in controlling the manifestations of ENL and cause partial or complete restoration of nerve function impairment, but they are associated with severe adverse effects and can lead to ENL recurrence upon tapering [20].Moreover, they are readily available over the counter, leading to self-medication, especially in India.

Thalidomide is a steroid-sparing drug effective in treatingENL. However, its usage is limited due to teratogenicity and neurotoxicity apart from availability [9]. Twenty-eight women were in the reproductive age group advised to use strict contraception. Thalidomide was prescribed after confirming that the women were negative for pregnancy. One out of 28 women was found to be pregnant after initiating thalidomide and was referred to an obstetrician–gynaecologist for further evaluation.The patient underwentelective termination of pregnancy at 11 weeks.According to the Medical Termination of Pregnancy (MTP) Act, 1971, in India, a provision is made to terminate the pregnancy when a woman received certain drugs, including thalidomideto prevent serious physical deformities in the newborn [21].The cases of thalidomide babies fall under section 3(2) (b) (ii) of MTP Act 1971 [22]. Though double contraception has been advised, it could not be ensured as the spouse has not returned for the review at every follow-up consultation, that might have resulted in an unplanned pregnancy. Hence, it is essential to emphasize adapting dual contraception methods in the wake of thalidomide embryopathy, which has social and personal negative implications.

Results published in the 1960s and 1980s on thalidomide through clinical trials showed good outcome in the treatment of ENL [23–25].These studies were done in a heterogeneous group of patients with small sample size and lack of standard criteria, which was a hurdle to evaluating the efficacy and adverse effects.

Dosage and duration of thalidomide in controlling ENL has been areas of uncertainty. However, a dose of 100 to 400 mg/day is routinely employed and adjusted according to severity [23]. In the present study, thalidomide was started at the dose of 300mg OD for a month and then reduced slowly by 100 mg each monthas a fixed-dose regimen for 2 months and then tapered depending on the clinical response of the patient. During this period, the patients were followed up regularly to ensure ENL has not deteriorated. In case of deterioration, patients were treated by increasing the thalidomide dose for a few weeks. This regimen was different from the study by Nidhi et al., [26] in which they started with 300mg daily for 1 to 2 months followed by a fixed dose of 200mg for 2 months and then 100mg for 2 months and then tapered based on patients marked clinical improvement.In a randomised study to compare the efficacy and safety of thalidomide over oral prednisolone in the treatment of ENL had shown excellent clinical response with thalidomide. They used thalidomide at a dose of 300 mg/day for 1 week, which was gradually reduced by 50 mg every 2 weeks [27].The variation in the clinical response is due to usage of different dosagesand duration of thalidomide in the aforementioned studies.

Arecent comparative study with prednisolone alone,thalidomide alone and prednisolone in combination with thalidomide or clofazimine in treatment of ENL found a lower recurrence rate in the thalidomide alone group, followed by the prednisolone plus thalidomide group during the 20-week treatment period [28]. Thalidomide induces faster clinical response (cutaneous and systemic) and is associated with fewer relapses, leading to complete recovery [27].

In compliant patients, thalidomide was given for the mean duration of approximately 11 months in our study(Table 2).The longer duration of treatment is due to steroid dependency mandatingtapering off the drug slowly to prevent recurrences. In one study, thalidomide was given for 10 years to control ENL episodes [29].However, ENL recurrence was observed in 16.2% of patients in this study,probably due to late initiation of thalidomide.A study reported a 6% (2/28) rate of recurrence post thalidomide [27].

**Fig 3:**
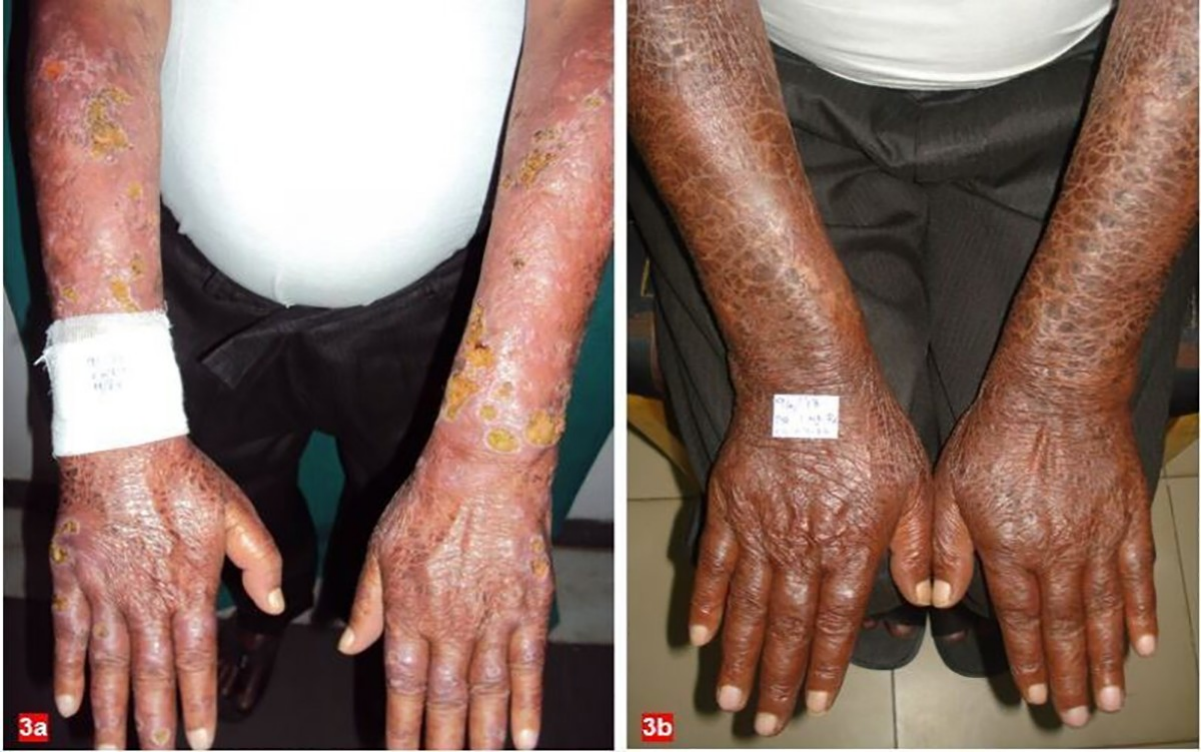
Clinical images of patient with ENL before (A) and after (B) thalidomide treatment.

It was observed that early thalidomide initiation (<6 months) and those with BI <4 responded well (Fig 3). In addition, those on MDT also showed a good response, which may be due to the presence of clofazimine in MDT, which has an anti-inflammatory effect.

**Fig 4:**
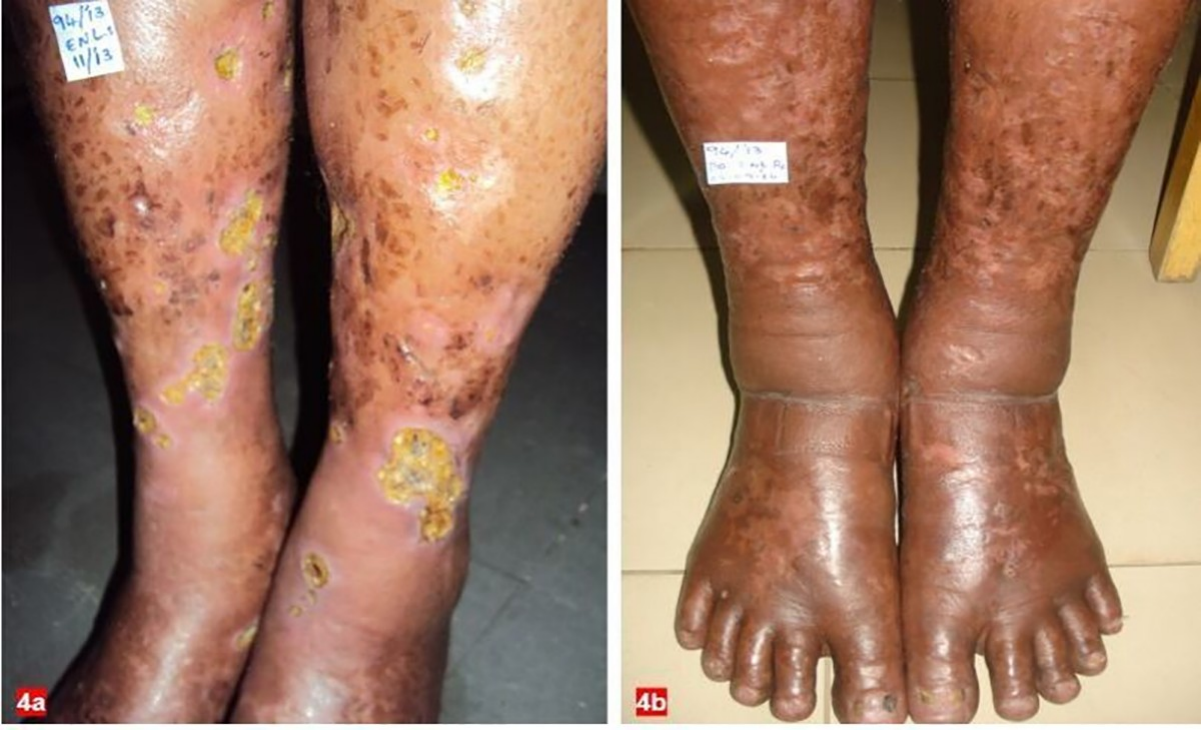
Clinical images of patient with ENL before (A) and after (B) thalidomide treatment.

Although teratogenicity is the most severe complication associated with thalidomide, persistent oedema of the face and extremities, urticaria, nausea, constipation, dryness of skin and mouth, increased sleep and appetite, hypothyroidism and peripheral neuropathy are also reported. In our study, pedal oedemawas the commonest adverse effect, noted in 73.5% patients (Fig 1) and is comparable with Parikh et al.where pedal oedema is the commonest side effect observed among 94 patients treated with thalidomide when given in a dose >100 mg/day [30]. Similarly, Grover et al. reported ankle oedema in 70% of patients on thalidomide treated for multiple myeloma [31].Walker et al. recognised that pedal oedema is a common complication of ENL, and hence, one can expect its increased incidence on thalidomide treatment [17] (Fig 4B). The frequency of side effects decreased during treatment.The exact mechanism by which thalidomide induces pedal oedema remainsunknown.However, it may be due to stasis of blood in the extremities and occurs at high doses. Pedal oedema in our study was transient and did not warrant thalidomide discontinuation. Drowsiness associated with thalidomide intake can be prevented by prescribing the drug at the bedtime or dividing the dose.

Peripheral neuropathy symptoms and deep vein thrombosis (DVT) were reported as the most common adverse events in a recent study on ENL treated with thalidomide [32]. The authors did not record the peripheral neuropathy as such, but associated symptoms noted wereparaesthesia, numbness, burning sensation, weakness and cramps,indicating the need for strict monitoring during treatment. Older patients who were on thalidomide for long duration and on a higher dosage had symptoms of neuropathy[33].The increased risk of DVT could bedue to a combination of certain chemotherapies and corticosteroids, along with thalidomide [34]. In our study, none of the patients had DVT (Fig 1) similar to that reported in the study by J. Darlong et al. [11].

Deaths reported in our study are based on clinical case records, and no post-mortem autopsies were performed. Two deaths were probably due to adrenal suppression resulting from long-term steroid use (Table 4). In our experience, treatment with thalidomide is found to be very effective in the management of ENL, and it also reduced the recurrences to a greater extent. It is observed in the study that the patients who were started on thalidomide therapy in the vast majority were steroid dependent.Thalidomide role as a first-linetreatment of ENL needs to be addressed through prospective multicentre studies, which would facilitate the formulation of policy guidelines.

Study limitations include the retrospective design; side-effects of thalidomide treatment were not documented systematically in patient notes but recorded only if they occurred.Thirty-four patients lost to follow-up. Despite these limitations, we could generate information on the treatment of ENL with thalidomide at outpatient clinics.

ENL is one of the commonest complications of leprosy, and corticosteroids remain the mainstay of treatment. Long-term usage of steroids poses the risk of adverse effects and dependency. The study enlightens the significant impact of thalidomide in controlling ENL in leprosy patients. It elicits a quick and effective response andcan be used as a steroid-sparing agent with minimal adverse effects. Thorough counselling to the patient and spouse/partner to follow dual contraceptives to avoid pregnancy is of utmost importance.We were largely successful in preventing pregnancies; however, one patient became pregnant during the study period.Timely identification of patients at risk of developing steroid dependence and instituting thalidomide can reduce morbidity and mortality among them. Clinicians must be awareof the occurrence of pedal oedema following the use of thalidomide, particularly at high doses, although this does not warrant drug discontinuation. It can be prescribed in outpatient settings.

## Supporting information

S1 Checklist Thalidomide STROBE checklist. (DOC)
