# Supplementary material for: Thalidomide in the treatment of erythema nodosum leprosum (ENL) in an outpatient setting: A five-year retrospective analysis from a leprosy referral centre in India
Source: PLoS Negl Trop Dis. 2020 Oct 9;14(10):e0008678. doi: 10.1371/journal.pntd.0008678 (PMC7577491; doi:10.1371/journal.pntd.0008678)
Supplement: S1 Checklist — (DOC) [file pntd.0008678.s001.doc]

STROBE Statement—Checklist of items that should be included in reports of ***cohort studies***

|  | Item No | Recommendation |
| --- | --- | --- |
| **Title and abstract** | 1 | (*a*) Indicate the study’s design with a commonly used term in the title or the abstract |
|  |  | (*b*) Provide in the abstract an informative and balanced summary of what was done and what was found  **“Title page and Abstract, page 1 and 2”** |
| Introduction | | |
| Background/rationale | 2 | Explain the scientific background and rationale for the investigation being reported  **“Introduction, paragraph 2-3, line 69 – 77”** |
| Objectives | 3 | State specific objectives, including any prespecified hypotheses  **“Introduction, paragraph 3, line 69 – 95”** |
| Methods | | |
| Study design | 4 | Present key elements of study design early in the paper  **“Methods, Patients, paragraph 1, line 103 – 104”** |
| Setting | 5 | Describe the setting, locations, and relevant dates, including periods of recruitment, exposure, follow-up, and data collection  **“Methods, Patients, paragraph 1, line 103 – 107”** |
| Participants | 6 | (*a*) Give the eligibility criteria, and the sources and methods of selection of participants. Describe methods of follow-up  **“Methods, patients, paragraph 1, line 103 – 117”** |
|  |  | (*b*) For matched studies, give matching criteria and number of exposed and unexposed |
| Variables | 7 | Clearly define all outcomes, exposures, predictors, potential confounders, and effect modifiers. Give diagnostic criteria, if applicable  **“Methods, Definitions, line 132 – 134”** |
| Data sources/ measurement | 8* | For each variable of interest, give sources of data and details of methods of assessment (measurement). Describe comparability of assessment methods if there is more than one group  **“Methods, paragraph 1, line 103 – 107; Definitions, line 121 – 134’’** |
| Bias | 9 | Describe any efforts to address potential sources of bias  **“Discussion, paragraph 12, line 310 – 312”** |
| Study size | 10 | Explain how the study size was arrived at  **“Methods, Patients, paragraph 1, line 103 – 104”** |
| Quantitative variables | 11 | Explain how quantitative variables were handled in the analyses. If applicable, describe which groupings were chosen and why  **“Methods, Definitions, line 121 – 134”** |
| Statistical methods | 12 | (*a*) Describe all statistical methods, including those used to control for confounding |
|  |  | (*b*) Describe any methods used to examine subgroups and interactions |
|  |  | (*c*) Explain how missing data were addressed |
|  |  | (*d*) If applicable, explain how loss to follow-up was addressed |
|  |  | (*e*) Describe any sensitivity analyses  **Methods, paragraph 4, line 148-158”** |
| Results | | |
| Participants | 13* | (a) Report numbers of individuals at each stage of study—eg numbers potentially eligible, examined for eligibility, confirmed eligible, included in the study, completing follow-up, and analysed |
|  |  | (b) Give reasons for non-participation at each stage |
|  |  | (c) Consider use of a flow diagram  “**Results, paragraph 1, line 166-168”** |
| Descriptive data | 14* | (a) Give characteristics of study participants (eg demographic, clinical, social) and information on exposures and potential confounders |
|  |  | (b) Indicate number of participants with missing data for each variable of interest |
|  |  | (c) Summarise follow-up time (eg, average and total amount)  **“Results, paragraph 1-3, line 162 – 191”** |
| Outcome data | 15* | Report numbers of outcome events or summary measures over time  **“Results, paragraph 5, line 200 – 205”** |
| Main results | 16 | (*a*) Give unadjusted estimates and, if applicable, confounder-adjusted estimates and their precision (eg, 95% confidence interval). Make clear which confounders were adjusted for and why they were included |
|  |  | (*b*) Report category boundaries when continuous variables were categorized |
|  |  | (*c*) If relevant, consider translating estimates of relative risk into absolute risk for a meaningful time period  **“Results, Paragraph 4-5, line 193 – 205”** |
| Other analyses | 17 | Report other analyses done—eg analyses of subgroups and interactions, and sensitivity analyses  **“Results, paragraph 5, line 193 – 204”** |
| Discussion | | |
| Key results | 18 | Summarise key results with reference to study objectives  **“Discussion, paragraph 7-9, line 277 – 300”** |
| Limitations | 19 | Discuss limitations of the study, taking into account sources of potential bias or imprecision. Discuss both direction and magnitude of any potential bias  **“Discussion, paragraph 12, line 319 – 322”** |
| Interpretation | 20 | Give a cautious overall interpretation of results considering objectives, limitations, multiplicity of analyses, results from similar studies, and other relevant evidence  **“Discussion,** **paragraph 13, line 324– 333”** |
| Generalisability | 21 | Discuss the generalisability (external validity) of the study results  **“Discussion, paragraph 11, line 312 – 318”** |
| Other information | | |
| Funding | 22 | Give the source of funding and the role of the funders for the present study and, if applicable, for the original study on which the present article is based  **LEPRA-BPHRC is core funded by LEPRA-UK. The funder had no role in the study design, data collection and analysis, decision to publish, or preparation of the manuscript.** |

*Give information separately for exposed and unexposed groups.

**Note:** An Explanation and Elaboration article discusses each checklist item and gives methodological background and published examples of transparent reporting. The STROBE checklist is best used in conjunction with this article (freely available on the Web sites of PLoS Medicine at http://www.plosmedicine.org/, Annals of Internal Medicine at http://www.annals.org/, and Epidemiology at http://www.epidem.com/). Information on the STROBE Initiative is available at http://www.strobe-statement.org.
